# Butterfly Pea (*Clitoria ternatea*), a Cyclotide-Bearing Plant With Applications in Agriculture and Medicine

**DOI:** 10.3389/fpls.2019.00645

**Published:** 2019-05-28

**Authors:** Georgianna K. Oguis, Edward K. Gilding, Mark A. Jackson, David J. Craik

**Affiliations:** Institute for Molecular Bioscience, The University of Queensland, St Lucia, QLD, Australia

**Keywords:** peptides, forage crop, anthocyanins, organic pesticide, butelase, medicinal plant

## Abstract

The perennial leguminous herb *Clitoria ternatea* (butterfly pea) has attracted significant interest based on its agricultural and medical applications, which range from use as a fodder and nitrogen fixing crop, to applications in food coloring and cosmetics, traditional medicine and as a source of an eco-friendly insecticide. In this article we provide a broad multidisciplinary review that includes descriptions of the physical appearance, distribution, taxonomy, habitat, growth and propagation, phytochemical composition and applications of this plant. Notable amongst its repertoire of chemical components are anthocyanins which give *C. ternatea* flowers their characteristic blue color, and cyclotides, ultra-stable macrocyclic peptides that are present in all tissues of this plant. The latter are potent insecticidal molecules and are implicated as the bioactive agents in a plant extract used commercially as an insecticide. We include a description of the genetic origin of these peptides, which interestingly involve the co-option of an ancestral albumin gene to produce the cyclotide precursor protein. The biosynthesis step in which the cyclic peptide backbone is formed involves an asparaginyl endopeptidase, of which in *C. ternatea* is known as butelase-1. This enzyme is highly efficient in peptide ligation and has been the focus of many recent studies on peptide ligation and cyclization for biotechnological applications. The article concludes with some suggestions for future studies on this plant, including the need to explore possible synergies between the various peptidic and non-peptidic phytochemicals.

## Introduction

*Clitoria ternatea*, commonly known as butterfly pea, is a perennial herbaceous plant from the Fabaceae family. It has recently attracted a lot of interest as it has potential applications both in modern medicine and agriculture, and as a source of natural food colorants and antioxidants. *C. ternatea* has long been cultivated as a forage and fodder crop, and early studies assessed the plant for these purposes (Reid and Sinclair, 1980; Barro and Ribeiro, 1983; Hall, 1985). Numerous field trials in Queensland, Australia, eventually led to the registry of *C. ternatea* cv. ‘Milgarra’ (Oram, 1992), the only cultivar in Australia that was released for grazing purposes (Conway and Doughton, 2005). Additionally, *C. ternatea* has been widely used in traditional medicine, particularly as a supplement to enhance cognitive functions and alleviate symptoms of numerous ailments including fever, inflammation, pain, and diabetes (Mukherjee et al., 2008).

In as early as the 1950s, studies on *C. ternatea* sought to elucidate its pharmacological activities, phytochemical composition and active constituents (Grindley et al., 1954; Piala et al., 1962; Kulshreshtha and Khare, 1967; Morita et al., 1976). The novel *C. ternatea* anthocyanins termed “ternatins” which render *C. ternatea* flowers with their vivid blue color, were first isolated in 1985 (Saito et al., 1985). Following further isolation and structural characterization of numerous other ternatins, the ternatin biosynthetic pathway was postulated a decade later (Terahara et al., 1998). In 2003, comparison of *C. ternatea* lines bearing different floral colors provided insights into the role of acylation on *C. ternatea* floral color determination (Kazuma et al., 2003a). The abundance of these unique anthocyanins alongside other secondary metabolites in *C. ternatea* makes the plant an ideal source of natural additives that can enhance the appearance and nutritive values of consumer products (Pasukamonset et al., 2016, 2017, 2018; Siti Azima et al., 2017). Although a number of recent studies has endeavored to elucidate the pharmacological activities of *C. ternatea* (Adhikary et al., 2017; Kavitha, 2018; Singh et al., 2018), the contribution of individual extract components on any bioactivity measured remains unknown.

**FIGURE 1:**
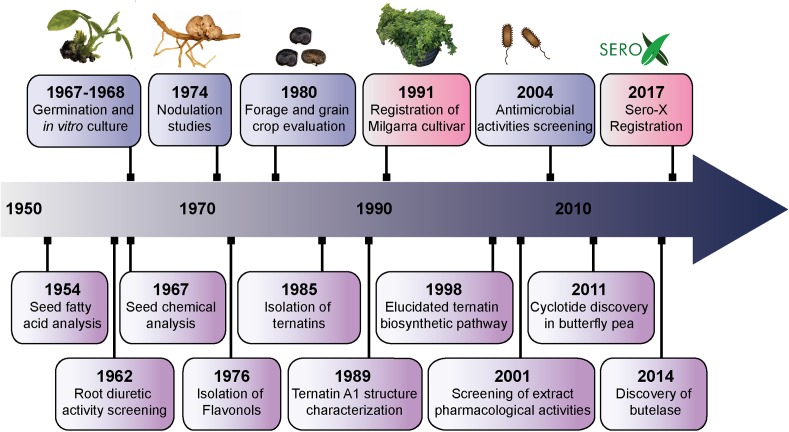
Timeline of the key studies and milestones on *Clitoria ternatea* research from the 1950s to the present. The biological (blue) and biochemical (purple) studies pursued from the 1950s to early 1970s characterized the properties of roots and seeds. Toward the end of the 1970s, researchers began to isolate and characterize the phytochemical compounds from *C. ternatea*. Ternatins, the anthocyanins that render *C. ternatea* its vivid blue color, were first isolated in 1985; and the structure of the largest of the ternatins, ternatin A1, was characterized in 1989. Further isolation and characterization of the ternatins in *C. ternatea* led to the elucidation of the ternatin biosynthetic pathway in 1998. Parallel to the studies that characterized the phytochemical composition of *C. ternatea*, were agricultural studies that evaluated *C. ternatea* as a forage and fodder crop. A series of field studies in Queensland, Australia lead the development and eventual release of the *C. ternatea* Milgarra cultivar in 1991. From 2001 to the present, studies have been determining the pharmacological activities and biological activities of *C. ternatea* extracts. In 2011, cyclotides, the circular insecticidal molecules which can also be used as scaffolds for peptide-based therapeutics, were discovered in *C. ternatea.* While cyclotides had previously been characterized in other angiosperm species, *C. ternatea* is to date, the only legume that is known to produce them. In 2014, butelase-1, the ligase that facilitates cyclization in *C. ternatea* cyclotides, was discovered and characterized. Cyclotides and the auxiliary enzymes, have applications both in modern medicine and agriculture. In 2017, Sero-X^®^ an eco-friendly insecticide made from *C. ternatea* extracts was registered for commercial use in Australia.

Figure 1 summarizes some of the key agricultural and biochemical studies conducted on *C. ternatea* from the 1950s to the present, providing a convenient timeline of discoveries. The corresponding references to the key studies and milestones are listed in Table 1. In recent years, the small circular defense molecules called cyclotides, in *C. ternatea* (Nguyen et al., 2011; Poth et al., 2011a,b; Nguyen et al., 2014) have fueled scientific innovations that may have impact in modern agriculture, biotechnology and medicine. In 2017, Sero-X^®^, a cyclotide-containing eco-friendly pesticide made from extracts of *C. ternatea*, was approved for commercial use in Australia^1^. In addition, the *C. ternatea* cyclotide processing enzyme, butelase-1, which is the fastest ligase known to date and is capable of ligating peptides across a vast range of sizes (26 to >200 residues), can potentially be used in the large scale synthesis of macrocycle libraries and peptide-based pharmaceuticals (Nguyen et al., 2014, 2015).

**Table 1:** Milestones in *Clitoria ternatea* studies.

Years	Milestones	References
1954	Seed fatty acid composition analyzed	Grindley et al., 1954
1962	Root diuretic properties screened	Piala et al., 1962
1967	Phytochemical composition of seeds analysis	Kulshreshtha and Khare, 1967
1967–1968	Germination studies and *in vitro* propagation	Mullick and Chatterji, 1967
1974	Nodulation pattern characterized	Oblisami, 1974
1976	Kaempferol-glycosides in leaves isolated	Morita et al., 1976
1980–1990	Forage and grain crop properties evaluated	Reid and Sinclair, 1980; Barro and Ribeiro, 1983; Hall, 1985
1985	Ternatins isolated from flowers	Saito et al., 1985
1989	Structure of Ternatin A1 determined	Terahara et al., 1989a
1991	Milgarra cultivar registered in Australia	Oram, 1992
1998	Ternatin biosynthetic pathway determined	Terahara et al., 1998
2000	Pharmacological activities of the extracts determined	Rai et al., 2001
2004	Antimicrobial properties characterized	Kelemu et al., 2004
2011	Cyclotides in *C. ternatea* discovered	Poth et al., 2011a,b
2014	Butelase discovered	Nguyen et al., 2014
2017	Sero-X^®^ registered	Innovate Ag, 2018^1^


*^1^https://innovate-ag.com.au/.*

### Plant Description

**FIGURE 2:**
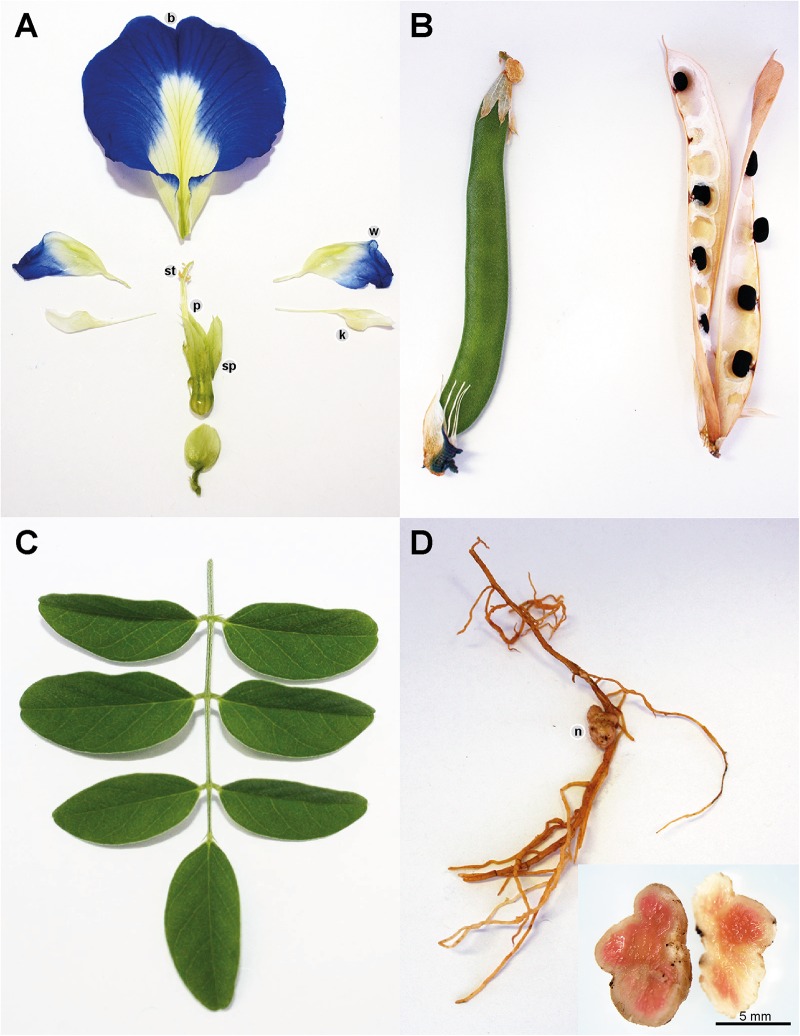
*Clitoria ternatea* **(A)** flower, **(B)** pods, **(C)** leaves, and **(D)** roots with nodules. The *C. ternatea* flower consists of the stamen (st), pistil (p), sepals (sp), and corollae. The corollae consist of five petals: one banner (b), two wings (w) and two keels (k). *C. ternatea* has pinnate compound leaves, flat and pointed pods and roots that produce nodules (n).

*Clitoria ternatea* produces pentamerous zygomorphic pea-shaped flowers with a tubular calyx consisting of five sepals which are fused about two thirds of their length. The showy corollae consists of five free petals, with one large and rounded banner, two wrinkled wings which are often half the length of the banner and two white keels which aid in protecting the floral organs (Cobley, 1956; Biyoshi and Geetha, 2012) (Figure 2A). The corollae are most often dark blue in color but may also occur in white and various blue and white shades in between (Morris, 2009; Biyoshi and Geetha, 2012). The diadelphous *C. ternatea* stamens consist of 10 filaments where nine are fused and one is free lying (Cobley, 1956; Biyoshi and Geetha, 2012). Attached to each filament is a pollen-bearing white anther, which consists of four lobes (Cobley, 1956; Pullaiah, 2000). *C. ternatea* produces a monocarpellary ovary bearing ten ovules (Pullaiah, 2000; Biyoshi and Geetha, 2012). Surmounting this is a long and thick style with a bent tip (Cobley, 1956; Biyoshi and Geetha, 2012). *C. ternatea* pods are narrow and flattened with pointy tips, and they typically contain around 10 seeds (Cobley, 1956) (Figure 2B). The seeds contain palmitic acid (19%), stearic acid (10%), oleic acid (51-52%), linoleic acid (17%) and linolenic acid (4%) (Grindley et al., 1954; Joshi et al., 1981). The caloric content of the seed is reported to be around 500 cal/100 g (Joshi et al., 1981). *C. ternatea* produces pinnate compound leaves that are obovate and entire with emarginate tips (Taur et al., 2010) (Figure 2C). The epidermis on both leaf surfaces consist of a single layer of cells protected by a thick cuticle and with trichome outgrowths (Taur et al., 2010). A layer of palisade cells, lignified xylem and paracytic stomata lie underneath the upper epidermis (Taur et al., 2010). *C. ternatea* produces an extensive deep-root system, which enables the plant to survive up to 7–8 months of drought (Cobley, 1956). The roots also produce large nodules for nitrogen fixation (Cobley, 1956) (Figure 2D).

### Taxonomy, Geographic Distribution and Habitat

The genus *Clitoria* occurs in tropical and subtropical environments across the globe. The number of subfamilial taxa remains unclear, and as in the case of *Clitoria*, the descriptions of species and citations of type specimens are noted as being incomplete or incorrect according to Fantz (1977). Thus, it is difficult to estimate species richness of the genus. Within *Clitoria*, three subgenera have been described and held as valid according to the monograph of *Clitoria*. Across all three subgenera, Fantz retains 58 species as valid, with numerous lower classifications of varieties and subspecies (Fantz, 1977).

**FIGURE 3:**
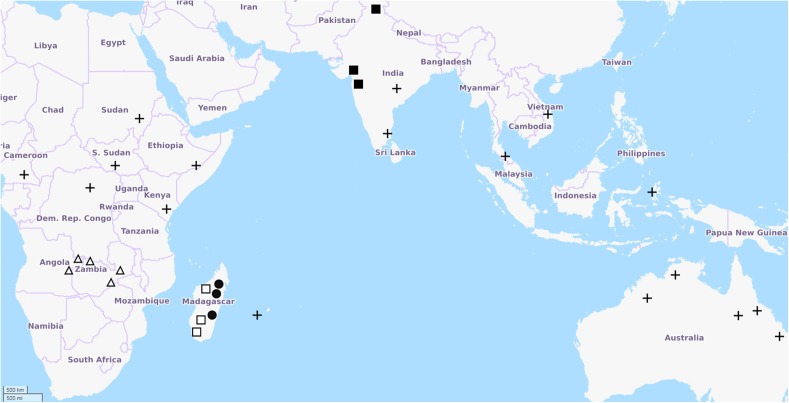
Distribution of *Clitoria* subgenus Clitoria species adapted from Fantz, 1977. Points of occurrence are approximate. Map data from Openstreetmap.org. Symbols represent: ◼ *C. biflora*, □ *C. heterophylla*, Δ *C. kaessneri*, • *C. lasciva*, + *C. ternatea*.

*Clitoria ternatea* is the holotype of *Clitoria* subgenus Clitoria, and represents the archetypical *Clitoria*. The etymology of the specific name is postulated to be from the island of Ternate in the Indonesian archipelago because it is from specimens from that location that Linnaeus produced the specific description. Ternate is not in the Indian Ocean but is instead in the Molucca Sea and in eastern Indonesia, lending ambiguity to the native range of the species. The distribution of all other taxa in subgenus Clitoria is restricted to Southern and Eastern Africa, India, Madagascar, and other islands of the Western Indian Ocean (Figure 3). The exact geographic origin of *C. ternatea* is thus difficult to determine, but we may infer from the center of diversity for subgenus Clitoria, that *C. ternatea* arose in or around the Indian Ocean and not the Pacific Ocean or South China Sea where it has been in use as a food coloring historically (Fantz, 1977; Staples, 1992). It is also entirely possible that the taxon we know as *C. ternatea* is an ancient hybrid of one or more members of the subgenus Clitoria that had subsequently been introduced to Southeast Asia. Testing of this synthetic origin hypothesis would require large scale genetics work on *C. ternatea* and related taxa like *Clitoria biflora*, *C. kaessneri*, *C. lasciva*, and *C. heterophylla*. Regardless of the specific geographical origin and evolutionary history of *C. ternatea*, the present day distribution of naturalized populations of *C. ternatea* is pantropical, as facilitated by key characteristics of the species: tolerance to drought conditions, non-reliance on specific pollinators because of self-pollination, and nitrogen fixation capability (Cobley, 1956; Staples, 1992; Conway et al., 2001). It is also possible to cultivate and maintain populations in subtropical regions (ex. Wee Waa NSW, located at -30.2, 149.433333).

The habitat of *C. ternatea* is open mesic forest or shrub land (personal observations of authors and records in the Australasian Virtual Herbrarium^2^). In Australia, the authors note that populations of *C. ternatea* occur in tropical regions in open areas where sunlight is plentiful due to a sparse canopy and in areas near where fresh water would collect such as the border of wetlands, small gullies, or at the base of rocky hillsides. When present, the plants are often vigorous and smother other vegetation.

### Growth and Propagation

Germination and establishment of *C. ternatea* is most favorable when the temperature is between 24–32°C, and when seeds are sown in moist soil at 2.5–5 cm deep and 20–30 cm apart (McDonald, 2002; Conway, 2005). Although *C. ternatea* can withstand arid conditions (Cobley, 1956), the plant grows best with ample moisture and rainfall (650–1250 mm) and when the temperature reaches 27°C or higher (Conway and Collins, 2005). Like most tropical legumes, *C. ternatea* is susceptible to frost damage (Conway and Collins, 2005). However, it can retain its leaves for as long as 7 days, and its woody parts typically recover (Conway and Collins, 2005).

Despite its hardy features, one of the impediments in propagating *C. ternatea* is its low seed germination rate. This problem has long been recognized as evident in a study conducted in 1967 (Mullick and Chatterji, 1967). The study showed that freshly harvested *C. ternatea* would not imbibe water and germinate (Mullick and Chatterji, 1967). On the other hand, storing the seeds for another 6 months promoted germination in 15–20% of the seeds (Mullick and Chatterji, 1967). Chemical scarification by means of soaking the seeds in boiling water or sulfuric acid was also found to promote *C. ternatea* seed germination (Cruz et al., 1995) where soaking the seeds in concentrated sulfuric acid for at least 10 min resulted in a reported 100% seed germination rate (Patel et al., 2016).

*In vitro* propagation can circumvent the unreliably low seed germination rate in *C. ternatea.* It can also be an alternative method for conserving and mass propagating *C. ternatea* lines with superior qualities. In 1968, a study determined the effects of adding ascochitine on the growth of *C. ternatea* embryos (Lakshmanan and Padmanabhan, 1968). That study reported that 60% of the embryos produced callus in both the upper and lower hypocotyl when 5–10 ppm ascochitine was added to the culture media. Numerous studies have since been conducted from 1990 to 2016 to determine the optimal plant hormone concentrations, basal media types and explant types for *C. ternatea in vitro* propagation (Table 2).

**Table 2:** Summary of published *Clitoria ternatea* *in vitro* propagation studies.

Hormone concentrations	Basal medium	Explants used	Results	References
–	MS	Mature embryo	Callus on seedling root	Lakshmanan and Dhanalakshmi, 1990
0.1 mg/L KN	MS	Mature embryo	Callus on seedling lateral root	Lakshmanan and Dhanalakshmi, 1990
0.5 mg/L KN	MS	Mature embryo	Callus on seedling root and hypocotyl; embryogenesis	Lakshmanan and Dhanalakshmi, 1990
0.5 mg/L KN + 0.5 mg/L IAA	MS	Mature embryo	Callus on seedling root; embryogenesis	Lakshmanan and Dhanalakshmi, 1990
1.12 mg/L BAP + 2.2 or 4.4 mg/L 2,4-D	MS	Excised root segments from aseptic seedlings	Organogenic callus	Shahzad et al., 2007
2.0 mg/L BAP + 1.0 mg/L NAA	DKW	Leaf explants from aseptic seedlings	Callus formation	Mohamed and Taha, 2011
1.0 mg/L NAA + 0.5 mg/L BAP + 40 mg/L 2iP	MS	Aseptic leaf explants encapsulated using 3% sodium alginate	Callus formation	Mahmad et al., 2016
0.56 - 2.25 mg/L BAP + 0.37 mg/L NAA	MS	Calli derived from excised root segments	Shoot proliferation	Shahzad et al., 2007
–	½ MS	Isolated shoot buds (0.2–0.5 cm in length) from mature embryo	Shoot proliferation	Lakshmanan and Dhanalakshmi, 1990
0.1–0.5 mg/L BAP	MS	Isolated shoot buds (0.2–0.5 cm in length) from mature embryo	Shoot proliferation	Lakshmanan and Dhanalakshmi, 1990
2.5 mg/L BAP + 0.25 mg/L NAA	MS	Axillary buds	Shoot proliferation	Mhaskar et al., 2011
2 mg/L BAP + 0.25 mg/L NAA	Semisolid MS	Nodal explants	Shoot proliferation	Rout, 2005
1.12 mg/L BAP	MS	Nodal explants	Shoot proliferation	Ismail et al., 2012
2.0 mg/L BAP	MS	Shoot tip, node, cotyledonary node explants	Shoot proliferation	Pandeya et al., 2010
0.5 mg/L GA	MS	Shoot tip, node, cotyledonary node explants	Shoot elongation	Pandeya et al., 2010
1.0 mg/L BAP	DKW	Leaf explants from aseptic seedlings	Shoot proliferation	Mohamed and Taha, 2011
4.5 mg/L BAP + 0.37 mg/L NAA	MS	Excised root segments from aseptic seedlings	Shoot proliferation	Shahzad et al., 2007
0.02 mg/L TDZ; 0.2 mg/L TDZ	MS	Cotyledonary node; nodal explants	Shoot proliferation	Mukhtar et al., 2012
0.1–0.5 mg/L IBA	MS	Isolated shoots (2.0–5.0 cm in length) proliferated from mature embryo	Rooting	Lakshmanan and Dhanalakshmi, 1990
0.1–0.5 mg/L IAA	MS	Isolated shoots (2.0–5.0 cm in length) proliferated from mature embryo	Rooting	Lakshmanan and Dhanalakshmi, 1990
0.25 mg/L NAA	½ MS (2% suc)	Directly regenerated shoots from nodal explants	Rooting	Rout, 2005
1.0 mg/L IBA	½ MS	Shoots derived from organogenic calli	Rooting	Shahzad et al., 2007
0.2–0.4 mg/L IBA	½ MS	Directly regenerated elongated shoots from nodal, cotyledonary nood and shoot tips	Rooting	Ismail et al., 2012; Mukhtar et al., 2012
0.56 mg/L NAA	MS	Directly regenerated shoots from axillary buds	Rooting	Mhaskar et al., 2011
Dipping in 250 mg/L IBA for 30 min	Soilrite	Elongated shoots	Rooting (*ex vitro*)	Pandeya et al., 2010
2.0 mg/L NAA	DKW	Leaf explants from aseptic seedlings	Rooting	Mohamed and Taha, 2011


*KN, kinetin; BAP, 6-benzylaminopurine; TDZ, thidiazuron; IAA, Indole-3-acetic acid; IBA, Indole-3-butyric acid; 2,4-D, 2,4-dichlorophenoxyacetic acid; NAA, 1-napthaleneacetic acid; GA, gibberellic acid; 2iP-N^*6*^, (2-Isopentenyl)adenine; MS, Murashige and Skoog medium (3% sucrose (suc) unless otherwise stated); DKW, Driver Kuniyuki Walnut medium (3% suc).*

With the optimal hormone concentrations supplemented in the basal medium, callus production was observed from mature *C. ternatea* embryos, leaf and root explants obtained from aseptic seedlings (Lakshmanan and Dhanalakshmi, 1990; Shahzad et al., 2007; Mohamed and Taha, 2011). In some instances, prolonged explant maintenance in the same callus induction medium led to embryoid production (Lakshmanan and Dhanalakshmi, 1990). Recently, a study described a protocol to produce encapsulated embryogenic callus from leaf explants using the optimal hormone concentrations and 3% sodium alginate (Mahmad et al., 2016). The study reported that more than 50% of the encapsulated explants stored at 4°C for 90 days survived (Mahmad et al., 2016). Studies showed that shoots can be regenerated from callus (Shahzad et al., 2007; Mahmad et al., 2016). Alternatively, shoots can also be induced and proliferated directly from different explant types such as isolated shoot buds (Lakshmanan and Dhanalakshmi, 1990), axillary buds (Mhaskar et al., 2011), shoot tips (Pandeya et al., 2010), leaf (Mohamed and Taha, 2011), and root (Shahzad et al., 2007) from aseptic seedlings, cotyledonary nodes (Pandeya et al., 2010; Mukhtar et al., 2012) and nodal explants (Rout, 2005; Pandeya et al., 2010; Ismail et al., 2012; Mukhtar et al., 2012). These *in vitro* grown *C. ternatea* shoots when subsequently placed in a medium supplemented with the optimal auxin concentrations produced roots *in vitro* (Lakshmanan and Dhanalakshmi, 1990; Rout, 2005; Shahzad et al., 2007; Mhaskar et al., 2011; Mohamed and Taha, 2011; Ismail et al., 2012; Mukhtar et al., 2012). Nevertheless, *ex vitro* root production was observed when elongated shoots were soaked in a concentrated auxin solution (Pandeya et al., 2010).

Moreover, a study has described propagation of *C. ternatea* via hairy root cultures (Swain et al., 2012b). Using the wild-type *Agrobacterium rhizogenes* strain A4T with the optimal culture conditions, a transformation frequency of as high as 85.8% was observed (Swain et al., 2012b). Compared to roots obtained from outdoor grown plants, *C. ternatea* hairy root cultures produced fourfold the amount of taraxerol, an anticancer triterpenoid compound that is naturally produced in *C. ternatea* roots (Swain et al., 2012a).

## Historical and Current Applications

### Agriculture

#### Fodder and Forage Crop

*Clitoria ternatea* has long been cultivated as a forage crop (Cobley, 1956), with yields reaching 17–29 tons/ha of palatable hay for cattle (Barro and Ribeiro, 1983; Abdelhamid and Gabr, 1993). This yield is on par with the established forage crop, alfalfa (*Medicago sativa*), and can potentially replace it in warm areas with low rainfall (Barro and Ribeiro, 1983). In Australia, *C. ternatea* has been cultivated predominantly in Queensland, due to its adaptability in the arid regions and persistence in heavy-textured farm lands (Hall, 1985). In 1991, the Queensland Department of Primary Industries, released the *C. ternatea* cv. ‘Milgarra’ mainly for grazing purposes (Oram, 1992). Milgarra is a composite of 21 introduced and naturalized *C. ternatea* lines that were grown for over three generations (Oram, 1992). As it is a composite cultivar, phenotypic variations are commonly observed in the field (Conway and Doughton, 2005).

Timing of harvest has been demonstrated to be important for maximizing dry matter content and digestibility of *C. ternatea* hay, with 45 days shown to be optimal (Mahala et al., 2012). Further increases in dry matter content have been reported if *C. ternatea* is pruned every 42 days at 20 cm (Colina et al., 1997), with dry matter yields of 1122 kg/ha reported. Compared to other legumes, animal feeds prepared from *C. ternatea* have consistently lower acid detergent fiber content. This low amount of acid detergent fiber increases energy density of the feed, and retains a high nitrogen content (Jones et al., 2000). Thus, feeds made from this plant have favorable nutritional characteristics compared to other legume forages. *C. ternatea* is also a great source of carotenoids with the carotenoid content of a 6-month old hay reaching 600 mg/kg dry matter (Barro and Ribeiro, 1983).

#### Nitrogen Fixation and Improvement of Soil Nutrient Content

*Clitoria ternatea* roots produce large round nodules (Cobley, 1956) (Figure 2D) known to house nitrogen-fixing bacteria, making the plant ideal for use in a crop rotation system. As early as the 1970s, studies were conducted to assess the nitrogen-fixing capacity of *C. ternatea* (Oblisami, 1974; De Souza et al., 1996). Nodulation was shown to be more favorably induced with a soil moisture content of around 25–45% with a light duration of 11–14 h and an intensity of 11–17 W/m^2^(Habish and Mahdi, 1983). Supplementing the soil with sulfur was also demonstrated as beneficial for nodule formation (Zaroug and Munns, 1980a). Several studies have reported the benefits of *C. ternatea* to soil health (De Souza et al., 1996; Dwivedi and Kumar, 2001; Kamh et al., 2002; Alderete-Chavez et al., 2011). Field trials conducted in Mexico reported that at 180 days post planting of *C. ternatea*, the organic matter, N, P, and K content of the soil all increased significantly (Alderete-Chavez et al., 2011). A similar study conducted in India reported that intercropping *C. ternatea* with the fodder crop *Setaria sphacelata* enriched the N content of the soil to an estimated 39.8 kg/ha (Dwivedi and Kumar, 2001). The results suggest that intercropping *C. ternatea* may potentially lead to a shorter fallow period requirement (Njunie et al., 2004).

When considering crop rotations, it is important to determine the cross nodulation capacity of nitrogen fixing *Rhizobium* species. One study showed that the *Rhizobium* species isolated from *C. ternatea*, cow pea and soybean are more compatible to each other than other legume species (Oblisami, 1974), while cross inoculation of *Rhizobium* sp. from *C. ternatea* and the legume species, *Phaseolus vulgaris*, *M. sativa*, and *Pisum sativum*, produced no nodules (Oblisami, 1974). These studies provide insights as to which legume species, when planted together with *C. ternatea*, are more likely to form nodules and thereby yield the most soil benefits. Another early study showed that the symbiotic efficiencies measured, based on *C. ternatea* dry matter yield, varied depending on the *Rhizobium* sp. strains tested (Zaroug and Munns, 1980b). A more recent study reported the isolation and identification of 11 rhizobial strains from *C. ternatea* grown in Thailand (Duangkhet et al., 2018). The 16s rDNA phylogenetic analysis revealed that ten of these isolates were *Bradyrhizobium elkanii* strains while the remaining isolate was a *Bradyrhizobium japonicum* strain. These *C. ternatea* *B. elkanii* strains were shown to promote better plant growth and induce higher nitrogen-fixing capacity than *B. elkanii* strains isolated from soybean (Duangkhet et al., 2018).

### Medicine

The popular use of *C. ternatea* in traditional medicine has stimulated researchers to elucidate the pharmacological activities of extracts obtained from various *C. ternatea* tissues. Numerous animal studies have reported that the extracts exhibit diuretic, nootropic, antiasthmatic, anti-inflammatory, analgesic, antipyretic, antidiabetic, antilipidemic, anti-arthritic, antioxidant, and wound healing properties. The results of the animal and in *in vitro* studies are summarized in Tables 3 and 4, respectively. Although these combined studies claim that *C. ternatea* extracts showcase a diverse range of pharmacological properties, many of these studies are preliminary and require more thorough investigation. In many instances the authors have attributed the extract activities to the presence of flavonols and anthocyanins, however, attempts to isolate and test individual components are limited. Indeed several components in *C. ternatea* extracts could be acting synergistically. For instance, cyclotides which have been reported to have immunosuppressive properties may contribute (Gründemann et al., 2012, 2013; Thell et al., 2016), as could the abundance of delphinidins (Sogo et al., 2015; Tani et al., 2017; Harada et al., 2018).

**Table 3:** Animal studies and clinical trial demonstrating the pharmacological activities of *Clitoria ternatea* extracts.

Tissue	Extraction solvent	Dosage (mg extract/kg body weight)	Administration	Experimental animals	Results	References
Roots	Ethanol	16 or 19	Single, oral	Dogs	No diuretic and natriuretic effects	Piala et al., 1962
Roots	Ethanol	4 or 6	Single, intravenous	Dogs	Increase in Na^+^ and K^+^, and decrease in Cl^-^ in the urine; no change in urine volume	Piala et al., 1962
Roots, aerial tissues	Ethanol	300 or 500	Daily for 7 d, oral	Wistar rats	Attenuated electric shock-induced amnesia; increase in acetylcholine content in the brain	Taranalli and Cheeramkuzhy, 2000
Roots	Water	50 or 100	Daily for 30 days, oral	7-day old Wistar rats	Improved memory retention and spatial learning 48 h and 30 days post treatment	Rai et al., 2001
Roots	Methanol	100, 200, or 400	Single, oral	Albino mice or Wistar rats	Nootropic, anxiolytic, antidepressant, anticonvulsant and anti-stress activities	Jain et al., 2003
Roots	Water	100	Daily for 30 days, oral	neonatal and adult Wistar rats	Higher hippocampal acetylcholine content in treated animals than their corresponding age group controls	Rai et al., 2002
Roots	Water	100	Daily for 30 days, oral	Adult Wistar rats	Increase in memory retention and spatial learning; increase in dendritic arborization	Rai et al., 2005
Leaves	Ethanol	200 or 400	Daily for 14 days, oral	Sprague Dawley rats	Decrease in acetylcholinesterase activity; decrease nitric oxide and lipid peroxide levels; increase in catalase, superoxide dismutase and glutathione levels	Talpate et al., 2014
Whole plant	–	3 g/kg	Fed for 60 days	Wistar rats	Protection of hippocampal cells through autophagy reduction	Raghu et al., 2017
Roots	Methanol	200 or 400	Single, oral	Wistar rats	Reduction in carrageenin-induced paw oedema and inhibition acetic acid-induced vascular permeability	Devi et al., 2003
Roots	Methanol	200, 300, or 400	Single, oral	Wistar rats	Reduction body temperature reduction	Parimaladevi et al., 2004
Leaves	Water, ethanol and petroleum ether	100–400	Single, oral	Wistar rats	Reduction in carrageenin-induced paw oedema; displayed analgesic activity determined by the tail flick method	Bhatia et al., 2014
Roots	Ethanol	100–150	Single, Intraperitoneal	Albino mice and Wistar rats	Decrease in leukocytosis and eosinophilia and inhibition of anaphylaxis in Wistar rats; protection from mast cell degranulation in albino mice	Taur and Patil, 2011
Flowers	Ethanol	400	Single, oral	Guinea pigs and albino mice	Reduction in histamine-induced dyspnoea in Guinea pigs; Reduction in coughing, lung inflammation, and decrease in white blood cell counts, interleukin and immunoglobulin G1 levels in albino mice	Singh et al., 2018
Leaves	Ethanol	400	Daily for 28 days, oral	Wistar rats	Reduction in the levels blood glucose, insulin, glycosylated hemoglobin, urea, creatinine and liver marker enzymes	Kavitha, 2018
Seeds, roots	Hydroalcohol	500	Single, oral	Sprague Dawley rats	Reduction in total serum cholesterol, triglyceride and very low density lipoprotein levels in rats with Poloxamer 407-induced hyperlipidemia	Solanki and Jain, 2010
Seeds, roots	Hydroalcohol	500	Daily for 7 days, oral	Sprague Dawley rats	Reduction in triglyceride and cholesterol levels	Solanki and Jain, 2010
Flowers	Methanol	50	Every other day for 24 days, oral	Male Swiss albino mice	Reduction in expression or release of enzymes, receptors or molecules implicated in inflammatory responses	Singh et al., 2018
Flowers^∗^	Aqueous	1–2 g in 400 mL water	Single, oral	Healthy adult males	Increase in plasma antioxidant capacity; decrease in postprandial sucrose and insulin levels; enhancement of postprandial antioxidant status	Chusak et al., 2018b


*^∗^Clinical trial.*

**Table 4:** *In vitro* studies demonstrating the pharmacological properties of *Clitoria ternatea* extract.

Extract	Concentration	*In vitro* assay	Results	References
Ethanolic floral extract	2.5–10 mg/mL	Extract addition to isolated adult goat tracheal tissue and guinea pig ileum dosed with histamine	Inhibition of histamine-induced contraction	Singh et al., 2018
Methanolic leaf extract	Six 2-fold dilution of 50 μg/ml	Hyaluronidase inhibition assay	Significant inhibition; IC_50_ = 18.08 ± 0.46 μg/ml	Maity et al., 2012
Methanolic leaf extract	Six 2-fold dilution of 50 μg/ml	Matrix metalloproteinase-1 inhibition assay (MMP-1)	Significant inhibition of MMP-1	Maity et al., 2012
Aqueous floral extract	400 μg/mL	Hemolytic and oxidation assays on canine erythrocytes	60% erythrocyte hemolysis protection after 6 h; decrease in lipid peroxidation and protein oxidation and increase in glutathione levels	Phrueksanan et al., 2014
Aqueous floral extract	1 and 2% (w/v) extract	Porcine α-amylase assay	Significant α-amylase inhibition, reduction in glucose release, hydrolysis index and glycemic index	Chusak et al., 2018a


#### Nootropic Activity

Several studies have reported improvement in cognitive performance when *C. ternatea* extracts were administered to experimental animals (Taranalli and Cheeramkuzhy, 2000; Rai et al., 2001; Jain et al., 2003). In one study, rats orally dosed with ethanolic extracts derived from *C. ternatea* roots or aerial tissues were shown to attenuate electric shock-induced amnesia better than the controls (Taranalli and Cheeramkuzhy, 2000). In a separate study, 7-day old neonatal rats orally dosed with aqueous *C. ternatea* root extract also showed improved memory retention and enhanced spatial learning performance 48 h and 30 days post treatment (Rai et al., 2001). Further investigations revealed that the brains of treated rats contained a significantly higher acetylcholine content than the controls (Taranalli and Cheeramkuzhy, 2000; Rai et al., 2002). A more recent study of the effects of *C. ternatea* leaf extracts on diabetic-induced cognitive decline showed that the acetylcholinesterase activity, total nitric oxide levels and lipid peroxide levels all significantly decreased upon treatment, whilst the catalase, superoxide dismutase and glutathione levels all significantly increased (Talpate et al., 2014). Another recent study showed that rats fed for 60 days with “medhya rasayana,” a mixture of crushed *C. ternatea* and jaggery (1:1), exhibited significant reduction in autophagy in the brain (Raghu et al., 2017). The treated and the control rats also differentially expressed genes implicated in autophagy regulation, nucleotide excision repair, homologous recombination, etc. The study suggested that *C. ternatea* protects the brain by affecting the autophagy directed pathway (Raghu et al., 2017).

#### Anti-inflammatory, Analgesic, and Antipyretic Activity

Extracts of *C. ternatea* roots and leaves have been reported to demonstrate anti-inflammatory, analgesic, and antipyretic activities (Devi et al., 2003; Parimaladevi et al., 2004; Bhatia et al., 2014; Singh et al., 2018). Oral administration of the methanolic root extracts and ethanolic floral extracts of *C. ternatea* was reported to significantly inhibit carrageenin-induced rat paw oedema and acetic acid-induced vascular permeability in rats (Devi et al., 2003; Singh et al., 2018). Results with an oral dosage of 400 mg extract per kg body weight were on par with a 20 mg/kg oral dosage of diclofenac sodium (Devi et al., 2003), a non-steroidal anti-inflammatory drug. In an antipyretic study, oral administration of *C. ternatea* methanolic root extracts significantly reduced the body temperature of Wistar rats that had yeast-induced elevated body temperature (Parimaladevi et al., 2004). This antipyretic activity of the extract was found to be comparable to paracetamol (Parimaladevi et al., 2004). More recently, *C. ternatea* leaf extracts have been implicated for use as an analgesic (Bhatia et al., 2014). In this study the established rat tail flick pain assay was used to determine the effects of pre-treatment with both ethanolic and petroleum *C. ternatea* extracts. A positive analgesic effect of *C. ternatea* leaf extracts was reported, comparable to diclofenac sodium (10 mg/kg) 1 h post treatment (Bhatia et al., 2014).

#### Antidiabetic Activity

Recently, *C. ternatea* leaf extracts have shown potential for use as an antidiabetic (Chusak et al., 2018b; Kavitha, 2018). Wistar rats orally dosed with 400 mg *C. ternatea* ethanolic leaf extract per kg of body weight per day for 28 days, had significantly lower levels of blood glucose, insulin, glycosylated hemoglobin, urea and creatinine than the diabetic control. Furthermore, the levels of liver enzymes (serum glutamate oxalate transaminase, serum glutamate pyruvate transaminase, lactate dehydrogenase, and alkaline phosphatase) in treated rats were lower than the diabetic control rats and were comparable to the normal control rats (Kavitha, 2018). More recent studies have focused on the effects of *C. ternatea* extracts on the glycemic response and antioxidant capacity in humans (Chusak et al., 2018b). A small scale clinical trial involving 15 healthy males revealed that when either 1 or 2 g of *C. ternatea* extract was ingested together with 50 g sucrose the resulting plasma glucose and insulin levels were suppressed (Chusak et al., 2018b). Furthermore the postprandial plasma antioxidant capacities of the subjects were also enhanced upon extract consumption.

#### Antioxidant Activity

The antioxidant properties of *C. ternatea* extracts are well documented (Phrueksanan et al., 2014; Sushma et al., 2015). One study demonstrated that *C. ternatea* extracts could protect canine erythrocytes from hemolysis and oxidative damage induced by 2,2′-azobis-2-methyl-propanimidamide dihydrochloride (AAPH) (Phrueksanan et al., 2014). Compared to the AAPH control, erythrocytes treated with 400 μg/mL of the *C. ternatea* extract had significantly lower levels of AAPH-induced lipid peroxidation and protein oxidation, and significantly higher levels of glutathione (Phrueksanan et al., 2014). In another study the antioxidant properties within a *C. ternatea* extract facilitated the production of magnesium oxide nanoparticles, materials which are increasingly being utilized for biomedical applications (Sushma et al., 2015).

### Pesticidal Activities

The anthelmintic and insecticidal activities, and the antimicrobial activities of *C. ternatea* extracts and several isolated protein and peptide components are summarized in Tables 5 and 6, respectively. These biological activities presumably evolved for host-defense purposes but can have potential applications both in agriculture and medicine. Further details on these activities are described in the following sections.

**Table 5:** Anthelmintic and insecticidal activities of *Clitoria ternatea*.

Biological activity	Organism	Results	References
Anthelmintic	*Meloidogyne incognita*	27 *C. ternatea* lines displayed varying degrees of resistance	Hasan and Jain, 1985
	*Meloidogyne incognita*	Methanolic leaf extract inhibited 93% of eggs from hatching	Kumari and Devi, 2013
	*Caenorhabditis elegans*	Significant toxicity of root extract on larvae	Gilding et al., 2015
	*Pheretima posthuma*	Ethanolic root extract increased mortality rate and number or paralyzed worms at 50 mg/mL	Khadatkar et al., 2008
	*Eisenia foetida*	Ethanolic and aqueous extract increased mortality and induced worm paralysis at 100 mg/mL	Salhan et al., 2011
Insecticidal	*Acanthoscelides obtectus*	1% w/w finotin application resulted to 100% larval mortality	Kelemu et al., 2004
	*Zabrotes subfasciatus*	5% w/w finotin application resulted to 100% larval mortality	Kelemu et al., 2004
	*Helicoverpa armigera*	Cter M cyclotide retarded larval growth in a dose dependent manner; 1 μmol/g diet induced larval mortality	Poth et al., 2011a
	*Helicoverpa* spp.	1–2% v/v oil-based extract resulted in larval mortality and reduced oviposition and larval feeding; detrimental effects against beneficial insects were not observed	Mensah et al., 2015


**Table 6:** Antimicrobial activities of *Clitoria ternatea*.

Biological activity	Organism	Extract/component	References
Antibacterial	*Bacillus cereus*	Ethanolic and aqueous leaf and callus extract	Shahid et al., 2009
	*Bacillus subtilis*	Ethanolic and aqueous leaf and callus extract	Shahid et al., 2009
	*Enterococcus faecalis*	Ethanolic leaf and callus extract	Shahid et al., 2009
	*Escherichia coli*	Cliotides T1, T4, T7, T15, T16, T19, and T20	Nguyen et al., 2011, 2016b
	*Klebsiella pneumoniae*	Cliotides T1 and T4	Nguyen et al., 2011, 2016c
	*Micrococcus luteus*	14.3 kDa seed protein	Ajesh and Sreejith, 2014
	*Pseudomonas aeruginosa*	Cliotides T1 and T4	Nguyen et al., 2011, 2016b
	*Staphylococcus aureus*	Ethanolic leaf and callus extract; ultrasound-assisted aqueous leaf and petal extract	Shahid et al., 2009; Anthika et al., 2015
	*Staphylococcus epidermidis*	Ethanolic leaf and callus extract	Shahid et al., 2009
	*Streptococcus pyogenes*	Ethanolic leaf and callus extract, aqueous leaf extract	Shahid et al., 2009
	*Streptococcus viridans*	Ethanolic leaf and callus extract	Shahid et al., 2009
	*Xanthomonas axonopodis*	Finotin	Kelemu et al., 2004
Antifungal	*Alternaria* sp.	14.3 kDa seed protein	Ajesh and Sreejith, 2014
	*Aspergillus flavus*	14.3 kDa seed protein	Ajesh and Sreejith, 2014
	*Aspergillus fumigatus*	14.3 kDa seed protein	Ajesh and Sreejith, 2014
	*Aspergillus niger*	14.3 kDa seed protein; methanolic leaf extract	Kamilla et al., 2009; Ajesh and Sreejith, 2014
	*Bipolaris oryzae*	Finotin	Kelemu et al., 2004
	*Colletotrichum gloeosporioides*	Finotin	Kelemu et al., 2004
	*Colletotrichum lindemuthianum*	Finotin	Kelemu et al., 2004
	*Candida albicans*	14.3 kDa seed protein	Ajesh and Sreejith, 2014
	*Candida parapsilosis*	14.3 kDa seed protein	Ajesh and Sreejith, 2014
	*Cryptococcus neoformans*	14.3 kDa seed protein	Ajesh and Sreejith, 2014
	*Cladosporium* sp.	14.3 kDa seed protein	Ajesh and Sreejith, 2014
	*Cryptococcus albidus*	14.3 kDa seed protein	Ajesh and Sreejith, 2014
	*Cryptococcus laurentii*	14.3 kDa seed protein	Ajesh and Sreejith, 2014
	*Curvularia* sp.	14.3 kDa seed protein	Ajesh and Sreejith, 2014
	*Fusarium oxysporum*	50% aqueous ethanolic leaf extract	Das and Chatterjee, 2014
	*Fusarium solani*	Finotin	Kelemu et al., 2004
	*Lasiodiplodia theobromae*	Finotin	Kelemu et al., 2004
	*Pyricularia grisea*	Finotin	Kelemu et al., 2004
	*Rhizoctonia solani*	Finotin	Kelemu et al., 2004
	*Rhizopus* sp.	14.3 kDa seed protein	Ajesh and Sreejith, 2014
	*Sclerotium* sp.	14.3 kDa seed protein	Ajesh and Sreejith, 2014


#### Anthelmintic Activity

The anthelmintic properties of *C. ternatea* have been reported in several studies (Hasan and Jain, 1985; Khadatkar et al., 2008; Salhan et al., 2011; Kumari and Devi, 2013; Gilding et al., 2015) (Table 5). Characterization of 27 homozygous *C. ternatea* lines showed that individual lines displayed different degrees of resistance against the parasitic root-knot nematode, *Meloidogyne incognita* (Hasan and Jain, 1985). The methanolic extract of *C. ternatea* was also found to inhibit 93% of *M. incognita* eggs from hatching (Kumari and Devi, 2013). In another study that utilized the model organism, *Caenorhabditis elegans*, *C. ternatea* extracts were found to effectively kill nematode larvae, with the root extracts showing greater lethality than the leaf extracts (Gilding et al., 2015). Two studies also reported *C. ternatea* activities against annelids (Khadatkar et al., 2008; Salhan et al., 2011). Using *Pheretima posthuma* as a test worm, one study showed that the ethanolic *C. ternatea* extract (50 mg/mL) caused significantly higher mortality rate and incidence of worm paralysis than piperazine citrate, a commonly used drug for controlling parasitic worms (Khadatkar et al., 2008). Similarly, using *Eisenia foetida* as a test worm, another study showed that the ethanolic and aqueous *C. ternatea* extract induced worm paralysis and mortality at 100 mg/mL (Salhan et al., 2011). However, compared to the commonly used antiparasitic drug levamisole, the rate of worm paralysis and death was significantly slower in the *C. ternatea* extracts (Salhan et al., 2011).

#### Insecticidal Activity

Proteins and peptides isolated from *C. ternatea* are reported to exhibit insecticidal properties (Kelemu et al., 2004; Poth et al., 2011a) (Table 5). One study reported 100% larval mortality when 1% w/w and 5% w/w of the purified *C. ternatea* protein (20 kDa), finotin, was applied to the bruchids *Acanthoscelides obtectus* and *Zabrotes subfasciatus*, respectively (Kelemu et al., 2004). Another study showed that when the *C. ternatea* cyclotide, Cter M, was incorporated in the diet of the lepidopteran species *Helicoverpa armigera*, larval growth retardation was observed in a dose dependent manner (Poth et al., 2011a). Larval mortality was observed at 1 μmol CterM peptide g^-1^ diet (Poth et al., 2011a).

Expanding on the initial findings of Poth et al. (2011a), additional studies have reported pesticidal activities of cyclotide extracts from *C. ternatea* (Gilding et al., 2015; Mensah et al., 2015) (Table 5). Gilding et al. (2015) showed that *C. ternatea* extracts permeabilized insect-like membrane lipids, with the shoot extracts exhibiting the greatest potency (0.31 μg/mL LC_50_). Another study reported that application of oil-based *C. ternatea* mixture (1–2% v/v) to transgenic and conventional cotton crops, resulted in *Helicoverpa* spp. larval mortality and reduced oviposition and larval feeding (Mensah et al., 2015). Detrimental effects of the extract against beneficial insects were not observed (Mensah et al., 2015), suggesting that *C. ternatea* extracts could provide the basis for eco-friendly natural insecticides.

#### Antimicrobial Activity

The antimicrobial properties of proteins isolated from *C. ternatea* have previously been described (Kelemu et al., 2004; Ajesh and Sreejith, 2014) (Table 6). The *C. ternatea* 20 kDa protein finotin demonstrated inhibitory activities over a wide range of plant fungal pathogens (Kelemu et al., 2004). Finotin also exhibited activities against the plant bacterial pathogen *Xanthomonas axonopodis* (Kelemu et al., 2004). Another study reported isolation of a 14.3 kDa protein from *C. ternatea* seeds (Ajesh and Sreejith, 2014) that exhibited activities against the human fungal pathogens, *Cryptococcus* spp. and *Candida* spp., and against a number of mold fungi (Ajesh and Sreejith, 2014). Studies also reported the antimicrobial properties of *C. ternatea* cyclotides against Gram-negative, but not Gram-positive, bacteria (Nguyen et al., 2011, 2016b).

Ethanol extract of *C. ternatea* outdoor grown leaves and calli inhibited the growth of the bacterial species *Staphylococcus* spp., *Streptococcus* spp., *Enterococcus faecalis*, and *Bacillus* spp. (Shahid et al., 2009). On the other hand, the antibacterial activities of the calli aqueous extract were only limited to *Bacillus* spp. and *Streptococcus pyogenes;* and activity of the leaf aqueous extract was limited to *Bacillus* spp. (Shahid et al., 2009). Furthermore, a recent study reported that the ultrasound-assisted aqueous extract of *C. ternatea* leaves and petals inhibited the growth of *Staphylococcus aureus* (Anthika et al., 2015). *C. ternatea* petals extracted for 30 min using ultrasound yielded the highest anthocyanin content and also displayed the highest antibacterial activity (Anthika et al., 2015).

The antifungal properties of *C. ternatea* have also been reported (Kamilla et al., 2009; Das and Chatterjee, 2014) (Table 6). Growth of the mold fungus *Aspergillus niger* was inhibited at a minimum inhibitory concentration of 0.8 mg/mL of the methanolic *C. ternatea* leaf extract (Kamilla et al., 2009). Scanning electron microscopy images from the study revealed that addition of the extract lead to conidial and hyphal collapse and distortion which is likely due to cell wall disruption (Kamilla et al., 2009). Another study reported that the 50% aqueous-ethanolic *C. ternatea* leaf extract inhibited the growth of *Fusarium oxysporum* and promoted the activities of amylase, protease and dehydrogenase in *P. sativum* seeds, enzymes that otherwise had low activities during *F. oxysporum* infestation (Das and Chatterjee, 2014).

## Phytochemical Composition

### Non-proteinaceous Components

#### Flavonols

As early as 1967, a study reported that *C. ternatea* seeds contain flavonol glycosides as well as phenolic aglycones, cinnamic acid, and a range of other compounds (Kulshreshtha and Khare, 1967). Nearly two decades later, Saito et al. (1985) reported the isolation of five *C. ternatea* flavonols, namely kaempferol, kaempferol 3-glucoside, kaempferol 3-robinobioside-7-rhamnoside, quercetin, and quercetin 3-glucoside (Saito et al., 1985). Subsequent studies reported the isolation of flavonol glycosides from *C. ternatea* leaves (Morita et al., 1976) and flowers (Kazuma et al., 2003a,b). With some exceptions, the identified flavonol glycosides were found in all *C. ternatea* lines bearing different floral colors (blue, mauve and white) (Kazuma et al., 2003a). For instance, myricetin 3-(2″-rhamnosyl-6″-malonyl)glucoside, myricetin 3-rutinoside and myricetin 3-glucoside were not detected in the *C. ternatea* line bearing mauve petals (Kazuma et al., 2003a). The flavonols isolated from *C. ternatea* are summarized in Table 7.

**Table 7:** Flavonol and anthocyanin content of *Clitoria ternatea*.

	Compound name	Tissue isolated	References
Flavonols	Kaempferol; Kaempferol 3-robinoside-7-rhamnoside	Blue flowers	Saito et al., 1985
	Kaempferol 3-glucoside;	Leaves, blue, mauve, and white flowers	Morita et al., 1976; Saito et al., 1985; Kazuma et al., 2003a,b
	Kaempferol 3-rutinoside; Kaempferol 3-neohesperidoside	Leaves, blue, mauve, and white flowers	Morita et al., 1976; Kazuma et al., 2003a,b
	Kaempferol-3-*O*-rhamnosyl-(1 → 2)-*O*-[rhamnosyl-1(1 → 6)]glucoside	Leaves	Morita et al., 1976
	Kaempferol 3-*O*-(2″-*O*-*a*-rhamnosyl-6″-*O*-malonyl)-*b*-glucoside; Kaempferol 3-(2^G^-rhamnosylrutinoside)	Blue, mauve, and white flowers	Kazuma et al., 2003a,b
	Quercetin	Blue flowers	Saito et al., 1985
	Quercetin 3-glucoside		Saito et al., 1985; Kazuma et al., 2003a,b; Adhikary et al., 2017
	Quercetin 3-*O*-(2″-*O*-*a*-rhamnosyl-6″-*O*-malonyl)-*b*-glucoside; Quercetin 3-rutinoside; Quercetin 3-(2^G^-rhamnosylrutinoside); Quercetin 3-neohesperidoside; Quercetin 3-glucoside;	Blue, mauve, and white flowers	Kazuma et al., 2003a,b
	Myricetin 3-neohesperidoside;	Blue, mauve, and white flowers	Kazuma et al., 2003a,b
	Myricetin 3-(2^G^-rhamnosylrutinoside)	Blue, mauve, and white flowers	Kazuma et al., 2003a
	Myricetin 3-*O*-(2″,6″-di-*O*-*a*-rhamnosyl)-β-glucoside; Myricetin 3-glucoside; Myricetin 3-rutinoside	Blue and white flowers	Kazuma et al., 2003a,b
Anthocyanins	Ternatin A1, A2, B1, D1	Blue flowers	Saito et al., 1985; Terahara et al., 1989a, 1990a; Kazuma et al., 2003a
	Ternatin B2, D2	Blue flowers	Saito et al., 1985; Terahara et al., 1989a, 1990a, 1996; Kazuma et al., 2003a
	Ternatin A3, B3-B4	Blue flowers	Terahara et al., 1996; Kazuma et al., 2003a
	Ternatin C1- C5, D3	Blue flowers	Terahara et al., 1998; Kazuma et al., 2003a
	Preternatin A3 and C4 (demalonylated analogs)	Mostly from young flowers	Terahara et al., 1998
	Delphinidin 3-*O*-(2″-*O*-*a*-rhamnosyl-6″-*O*-malonyl)-β-glucoside; Delphinidin 3-(6″-malonyl) glucoside; Delphinidin 3-neohesperidoside; Delphinidin 3-glucoside	Mauve flowers	Kazuma et al., 2003a
	3-*O*-(6″″-*O*-malonyl)- β-glucoside-3″-*O*-β-glucoside	Blue flowers	Kazuma et al., 2004


#### Anthocyanins

In 1985, six acylated anthocyanins were isolated from blue *C. ternatea* flowers that were all derivatives of delphinidin 3,3′,5′-triglucoside (Saito et al., 1985). The chemical properties of the acylated *C. ternatea* delphinidins, which were named ternatins, were further elucidated in subsequent studies (Terahara et al., 1989a, 1990a,b). In 1989, the structure of the largest isolated blue anthocyanin, ternatin A1, was determined (Terahara et al., 1989a). The study also showed that not only was ternatin A1 the largest, it was also one of the most stable in neutral solution (Terahara et al., 1989a). The structure of ternatins A2 (Terahara et al., 1990c), B1 (Kondo et al., 1990), B2 (Terahara et al., 1996), D1 (Terahara et al., 1989b), and D2 (Terahara et al., 1996) were elucidated shortly after.

**FIGURE 4:**
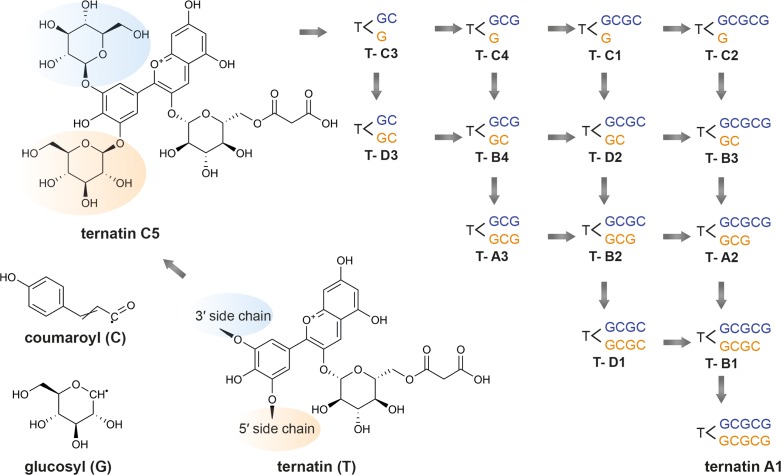
Proposed ternatin biosynthetic pathway. Adapted from Terahara et al. (1998). Beginning with ternatin C5 (PubChem CID 10843319), ternatin A1 (PubChem CID 16173494) can be produced through the addition of four *p-*coumaroyl (C) and four glucosyl moieties (G) at the 3′ sidechain (in blue) and 5′ sidechain (in orange). The other ternatins are products of the intermediate steps.

Subsequent studies isolated and determined the structures of several other novel ternatins isolated from *C. ternatea*: ternatins A3, B3–B4, C1–C5, D3, and preternatins A3 and C4 (Terahara et al., 1996, 1998) (Table 7). Terahara et al. (1998) observed that lower molecular weight ternatins are more abundant in young flowers while higher molecular weight ternatins are more prevalent in mature flowers. The authors proposed that ternatin A1 is the final compound, and the other ternatins are intermediate products (Terahara et al., 1998). Starting with ternatin C5, production of ternatin A1 can be achieved via four *p-*coumaric acid acylation steps and four glucosylation steps (Terahara et al., 1998). The biosynthetic pathway of ternatin A1 is summarized in Figure 4 (Terahara et al., 1998). The key enzymatic steps and the biosynthetic pathway to produce ternatin C5 from delphinidin was elucidated in 2004 (Kazuma et al., 2004).

A 2003 study compared the anthocyanin contents of *C. ternatea* lines bearing different floral colors (Kazuma et al., 2003a). The study showed that white *C. ternatea* flowers do not produce anthocyanins. Furthermore, unique to the mauve *C. ternatea* flowers, is the accumulation delphinidins lacking the 3′ and 5′ (polyacelated) glucosyl group substitutions (Kazuma et al., 2003a). The study concluded that glucosylation of delphinidins at these positions are crucial to the production of *C. ternatea* flowers (Kazuma et al., 2003a).

#### Other Non-proteinaceous Components

The pentacyclic triterpenoids, taraxerol and taraxerone, were isolated from *C. ternatea* roots in the 1960s (Banerjee and Chakravarti, 1963, 1964). Realizing the potential of *C. ternatea* as a source of taraxerol, in 2008, a method was developed for the routine quantification of the content in *C. ternatea* extracts of this medicinal compound (Kumar et al., 2008). In 2012, *in vitro* propagated hairy root cultures were sought as alternative to *in vivo* grown roots as source of taraxerol (Swain et al., 2012a). In 2016, in addition to taraxerol, novel norneolignans, clitorienolactones A-C, were isolated from *C. ternatea* roots (Vasisht et al., 2016). *C. ternatea* floral extracts also contain other types of flavonoids, including rutin (flavone), epicatechin (flavanol) and other polyphenolic acids (gallic acid, protocatechuic acid, and chlorogenic acid) (Siti Azima et al., 2017).

### Proteinaceous Components

**FIGURE 5:**
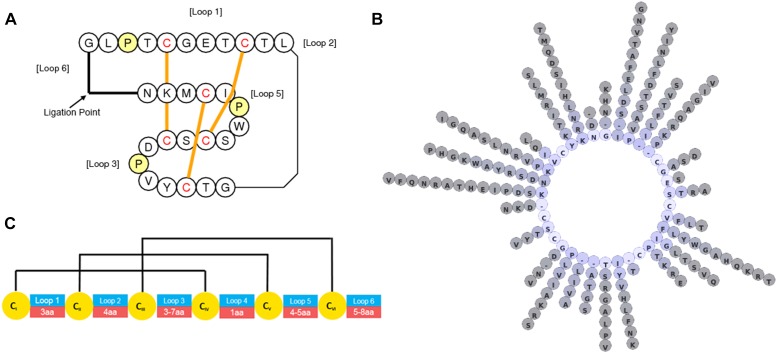
Graphical representations of *C*. *ternatea* cyclotide structure and diversity. **(A)** Topological depiction of Cter M displaying the position and threading of disulfide bonds. **(B)** Diversity of residues at non-cystine positions of 74 sequence defined *C*. *ternatea* cyclotides. **(C)** Loop length diversity and disulfide connectivity map of *C*. *ternatea* cyclotides.

In general, there has traditionally been a greater focus in phytochemical studies on the non-protein components of plants and *C. ternatea* is no exception. However, over the last decade, with advances in nucleic acid sequencing and mass spectroscopic peptide and protein characterization techniques there is now much more focus on proteinaceous components, particularly in the characterization of peptides and proteins implicated in plant defense. Of the known *C. ternatea* phytochemical components implicated in defense, a class of peptides known as cyclotides is particularly noteworthy (Nguyen et al., 2011; Poth et al., 2011a,b). These peptides mature into cyclic molecules of ∼30 aa from linear precursors through an enzymatic transpeptidation reaction of the peptide backbone. Cyclotides contain three disulfide bonds that form a knot (Figure 5A), similar to configurations seen in linear knottins cataloged across diverse taxa (Gelly et al., 2004). Together, the cyclic and knotted nature of cyclotides makes them highly stable in conditions that would otherwise facilitate peptide degradation (Craik et al., 1999). Unlike linear knottins, which are found across multiple kingdoms of life, cyclotides are restricted to relatively few taxa in Viridiplantae, namely the dicotyledon angiosperms (Gruber et al., 2008). Searching all Viridiplantae sequences for cyclotides using the widely distributed program tblastn, has highlighted the restriction of cyclotides and linear non-cyclotide-like sequences to a handful of plant families discussed below (Altschul et al., 1990).

Despite reports of cyclotide-like sequences in the Poaceae, none of the described sequences have been shown to exist as cyclic molecules *in planta*, thus failing the definition of the term cyclotide. The taxonomic distribution of cyclotides is often disjointed in a taxonomic group; for instance the taxonomically sparse occurrence of cyclotides observed in the Rubiaceae (Gruber et al., 2008; Koehbach et al., 2013) contrasts with the ubiquitous occurrence of cyclotides in all species tested far-off the Violaceae family (Burman et al., 2015; Göransson et al., 2015). Within the Fabaceae, *C. ternatea* is the only family member in which cyclotides have been observed despite examination of diverse Fabaceae, including other species of *Clitoria* (Gilding et al., 2015). Cyclotides are therefore one of the most interesting proteinaceous components of *C. ternatea*. That they are processed from genetically encoded precursor proteins opens opportunities for detecting them either or both as nucleic acid or peptide sequences.

#### Gene and Transcript Characterization

RNA-seq experiments to define the transcripts that encode for cyclotides have been performed by several groups. The resulting transcriptomes have allowed the cataloging of at least 74 cyclotide sequences (Table 8) which exhibit high levels of diversity at loops intervening the conserved Cys residues (Figure 5B,C) (Gilding et al., 2015; Nguyen et al., 2016b). All of the precursors observed have singleton cyclotide domains similar to that observed in *Petunia* x *hybrida* (Solanaceae), whereas cyclotide precursors from the Cucurbitaceae, Rubiaceae, and Violaceae families possess multiple cyclotide domains (Table 9) (Felizmenio-Quimio et al., 2001; Mylne et al., 2011; Poth et al., 2011a; Koehbach and Gruber, 2015; Park et al., 2017). Unlike precursors from the Cucurbitaceae, Rubiaceae, Solanaceae, and Violaceae, *C. ternatea* cyclotides are encoded in albumin-1 genes (Poth et al., 2011a).

**Table 8:** Cyclotides in *Clitoria ternatea*.

Cyclotide	Other name	Sequence	References
Cliotide T1		GIPCGESCVFIPCITGAIGCSCKSKVCYRN	Nguyen et al., 2011
Cliotide T2		GEFLKCGESCVQGECYTPGCSCDWPICKKN	Nguyen et al., 2011
Cliotide T4	Cter P	GIPCGESCVFIPCITAAIGCSCKSKVCYRN	Nguyen et al., 2011; Gilding et al., 2015
Cliotide T6		SIPCGESCVYIPCLTTIVGCSCKNSVCYSN	Nguyen et al., 2011
Cliotide T8		GIPCGESCVFIPCISSVVGCSCKSKVCYNN	Nguyen et al., 2011
Cliotide T9		GIPCGESCVFIPCITTVVGCSCKNKVCYNN	Nguyen et al., 2011
Cliotide T10	Cter 27	GIPCGESCVYIPCTVTALLGCSCKDKVCYKN	Nguyen et al., 2011; Gilding et al., 2015
Cliotide T11	Cter 21	GIPCGESCVFIPCTITALLGCSCKDKVCYKN	Nguyen et al., 2011; Gilding et al., 2015
Cliotide T12		GIPCGESCVFIPCITGAIGCSCKSKVCYRD	Nguyen et al., 2011
Cliotide T13	Cter 23	DTTPCGESCVWIPCVSSIVGCSCQNKVCYQN	Nguyen et al., 2011; Gilding et al., 2015
Cliotide T14		DTIPCGESCVWIPCISSILGCSCKDKVCYHN	Nguyen et al., 2016b
Cliotide T15	Cter24	GLPICGETCFKTKCYTKGCSCSYPVCKRN	Nguyen et al., 2011; Gilding et al., 2015
Cliotide T16		GSVIGCGETCLRGRCYTPGCTCDHGICKKN	Nguyen et al., 2016b
Cliotide T17		GTVPCGESCVFIPCITGIAGCSCKNKVCYLN	Nguyen et al., 2016b
Cliotide T18	Cter 6	GLPICGETCFTGTCYTPGCTCSYPVCKKN	Nguyen et al., 2011; Gilding et al., 2015
Cliotide T19a	Cter26	GSVIKCGESCLLGKCYTPGCTCSRPICKKN	Nguyen et al., 2011; Gilding et al., 2015
Cliotide T19b		GSVIKCGESCLLGKCYTPGCTCSRPICKKD	Nguyen et al., 2011
Cliotide T20		GSAIRCGESCLLGKCYTPGCTCDRPICKKN	Nguyen et al., 2016b
Cliotide T21	Cter 17	DLQCAETCVHSPCIGPCYCKHGLICYRN	Nguyen et al., 2011; Gilding et al., 2015
Cliotide T22^α^		ARIPCGESCVWIPCTITALVGCACHEKVCYKS	Nguyen et al., 2016b
Cliotide T23^∗^		GFPCGESCVFIPCTVTALLGCSCKDKVCYKN	Nguyen et al., 2016b
Cliotide T25^∗^		GSIRCGERCLLGRCHRPGCTCVRRICRRN	Nguyen et al., 2016b
Cliotide T26^∗^		GFICGESCVYIPCITALLGCSCSNQICSKN	Nguyen et al., 2016b
Cliotide T27^∗^		GVIPCGESCVFIPCITGAIGCSCKSKVCYRN	Nguyen et al., 2016b
Cliotide T30^∗^		GDPLKCGESCFAGKCYTPGCTCSRPICKKN	Nguyen et al., 2016b
Cliotide T31^∗^		GDPLKCGESCFAGKCYTPGCTCDRPICKKN	Nguyen et al., 2016b
Cliotide T32^∗^		GDLFKCGETCFGGTCYTPGCSCDYPICKNN	Nguyen et al., 2016b
Cliotide T37^α^		VDGFCLETCVILPCFSSVAGCYCHGSTCMRG	Nguyen et al., 2016b
Cliotide T38^α^		KIPCGESCVWIPCFTSAFGCYCQSKVCYHS	Nguyen et al., 2016b
Cliotide T42^∗^		DIPCGSTCLHVKCIPPCYCKNKVLCYRN	Nguyen et al., 2016b
Cliotide T47^β^		XIPCGESCVYLPCLTTIVGCSCKNNVCYTN	Nguyen et al., 2016b
Cliotide T48^β^		XCGESCVFLPCFIIPGCSCKDKVCYLN	Nguyen et al., 2016b
Cliotide T49^β^		NSAFCGETCVLGTCYTPDCSCKAVVCX	Nguyen et al., 2016b
Cliotide T50^β^		GVSWICDQTCLMQGKCYRSGCTCDRPX	Nguyen et al., 2016b
Cliotide T51^β^		GVPLCGETCFMGSCYTPGCSCDAVX	Nguyen et al., 2016b
Cliotide T52^β^		GDALKCGETCFGGTCYTPGCSX	Nguyen et al., 2016b
Cliotide T53^β^		GSSIVTCGETCLRGRCYTPGCX	Nguyen et al., 2016b
Cter 1^∗^	Cliotide T35	GLPICGETCFGGTCNTPNCVCDPWPICTNN	Gilding et al., 2015; Nguyen et al., 2016b
Cter 10^∗^	Cliotide T34	SYIPCGESCVYIPCTVTALLGCSCSNKVCYKN	Gilding et al., 2015
Cter 11^∗^	Cliotide T24	GSIRCGERCLLGRCHRPGCTCIRRICRRN	Gilding et al., 2015; Nguyen et al., 2016b
Cter 12^∗^		NTAFCGETCVLGTCYTPDCSCKAVVCIKN	Gilding et al., 2015
Cter 13^∗^		GSAIRCGERCLLGRCHRPGCTCIRRICRRN	Gilding et al., 2015
Cter 14^∗^	Cliotide T40	GIPCGESCVFIPCTITALLGCSCKSKVCYKN	Gilding et al., 2015; Nguyen et al., 2016b
Cter 15^∗^		GIPCGESCVFIPCTVTALLGCSCKSKVCYKN	Gilding et al., 2015
Cter 16^∗^	Cliotide T28	GGSIPCGESCVFLPCFLPGCSCKSSVCYLN	Gilding et al., 2015; Nguyen et al., 2016b
Cter 18^∗^	Cliotide T43	DLICSSTCLHTPCKASVCYCKNAVCYKN	Gilding et al., 2015; Nguyen et al., 2016b
Cter 19^∗^		SIPCGESCVYIPCLTTIVGCSCKSNVCYSN	Gilding et al., 2015
Cter 2^∗^	Cliotide T29	GDPLKCGESCFAGKCYTPGCTCEYPICMNN	Gilding et al., 2015
Cter 20^∗^		GVIPCGESCVYLPCLTTIVGCSCKNNVCYTN	Gilding et al., 2015
Cter 22^∗^		NTAFCGETCVLGTCYTPDCSCTAIVCIKN	Gilding et al., 2015
Cter 25^∗^	Cliotide T41	GNPIVCGETCFFQKCYTPGCSCDAVICTNN	Gilding et al., 2015; Nguyen et al., 2016b
Cter 28^∗^	Cliotide T36	GVIPCGESCVWIPCISAAIGCSCKKNVCYRN	Gilding et al., 2015; Nguyen et al., 2016b
Cter 29^∗^	Cliotide T44	GALCDERCTYVPCISAARGCSCNIHRVCSMN	Gilding et al., 2015; Nguyen et al., 2016b
Cter 3^∗^		GAFCGETCVLGTCYTPDCSCKAVVCIKN	Gilding et al., 2015
Cter 30^∗^	Cliotide T45	GFPICGETCFKTKCYTPGCSCSYPVCKKN	Gilding et al., 2015; Nguyen et al., 2016b
Cter 31^∗^	Cliotide T46	DLQCAETCVHSPCIGPCYCKHGVICYKN	Gilding et al., 2015; Nguyen et al., 2016b
Cter 32^∗^		KIPCGESCVWIPCISSILGCSCKDKVCYHN	Gilding et al., 2015
Cter 33^∗^		GDLFKCGETCFGGTCYTPGCSCDYPICKKN	Gilding et al., 2015
Cter 34^∗^	Cliotide T33	GFNSCSEACVYLPCFSKGCSCFKRQCYKN	Gilding et al., 2015; Nguyen et al., 2016b
Cter 35^∗^		GAFCGETCVLGTCYTPGCSCAPVICLNN	Gilding et al., 2015
Cter 36^∗^		GSPTCGETCFGGTCYTPNCVCDPWPICTKN	Gilding et al., 2015
Cter 37^∗^		GSPTCGETCFGGTCYTPGCVCDPWPICTKN	Gilding et al., 2015
Cter 4^∗^	Cliotide T39	GDPLACGETCFGGTCYTPGCVCDPWPICTKN	Gilding et al., 2015; Nguyen et al., 2016b
Cter 5^∗^		GEFLKCGESCVQGECYTPGCSCDYPICKNN	Gilding et al., 2015
Cter 7^∗^		GDPFKCGESCFAGKCYTPGCTCEYPICMNN	Gilding et al., 2015
Cter 8^∗^		GSAFCGETCVLGTCYTPDCSCKAVVCIKN	Gilding et al., 2015
Cter 9^∗^		GIPCGESCVYIPCTVTALLGCSCRDKVCYKN	Gilding et al., 2015
Cter A		GVIPCGESCVFIPCISTVIGCSCKNKVCYRN	Poth et al., 2011a,b
Cter B		GVPCAESCVWIPCTVTALLGCSCKDKVCYLN	Poth et al., 2011b
Cter C		GVPCAESCVWIPCTVTALLGCSCKDKVCYLD	Poth et al., 2011b
Cter D		GIPCAESCVWIPCTVTALLGCSCKDKVCYLN	Poth et al., 2011b
Cter E		GIPCAESCVWIPCTVTALLGCSCKDKVCYLD	Poth et al., 2011b
Cter F		GIPCGESCVFIPCISSVVGCSCKSKVCYLD	Poth et al., 2011b
Cter G		GLPCGESCVFIPCITTVVGCSCKNKVCYNN	Poth et al., 2011b
Cter H		GLPCGESCVFIPCITTVVGCSCKNKVCYND	Poth et al., 2011b
Cter I		GTVPCGESCVFIPCITGIAGCSCKNKVCYIN	Poth et al., 2011b
Cter J		GTVPCGESCVFIPCITGIAGCSCKNKVCYID	Poth et al., 2011b
Cter K		HEPCGESCVFIPCITTVVGCSCKNKVCYN	Poth et al., 2011b
Cter L		HEPCGESCVFIPCITTVVGCSCKNKVCYD	Poth et al., 2011b
Cter M	Cliotide T3	GLPTCGETCTLGTCYVPDCSCSWPICMKN	Nguyen et al., 2011; Poth et al., 2011a
Cter N		GSAFCGETCVLGTCYTPDCSCTALVCLKN	Poth et al., 2011b
Cter O		GIPCGESCVFIPCITGIAGCSCKSKVCYRN	Poth et al., 2011a
Cter Q	Cliotide T5	GIPCGESCVFIPCISTVIGCSCKNKVCYRN	Nguyen et al., 2011; Poth et al., 2011a
Cter R	Cliotide T7	GIPCGESCVFIPCTVTALLGCSCKDKVCYKN	Nguyen et al., 2011; Poth et al., 2011a
Cterneg_C1^α^		GSPLLRGETCVLQTCYTPGCSCTIAICLNN	Gilding et al., 2015


*^∗^No mass spec data available; ^α^predicted to be non-cyclic; ^β^low confidence sequences.*

**Table 9:** Characteristics of cyclotide gene precursors.

Taxonomy		Precursor characters			
Family	Exemplary species	Signal peptide to cyclotide N-terminal junction present	N-terminal pre- sequence present	Multimeric or singleton cyclotide domains present	C-terminal sequence type
Cucurbitaceae	*Momordica cochinchinensis*	N	Y	Multimeric	None, except final repeat is acyclic
Fabaceae	*Clitoria ternatea*	Y	N	Singleton	Linker and albumin-1 a-chain domain
Rubiaceae	*Oldenlandia affinis*	N	Y	Both	Short, ∼5 aa
Solanaceae	*Petunia* x *hybrida*	N	Y	Singleton	Short, ∼5 aa
Violaceae	*Viola tricolor*	N	Y	Both	Short, ∼5 aa


**FIGURE 6:**
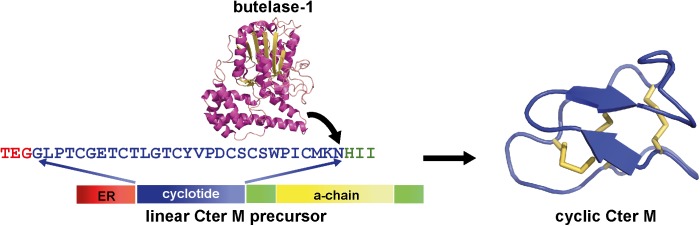
Structure of a Fabaceae albumin-1 cyclotide prepropeptide exemplified by Cter M. The linear Cter M precursor consists of the endoplasmic reticulum (ER) signal (red), the cyclotide domain (blue) which replaces the typical Fabaceae albumin-1 b-chain, the a-chain (yellow) and the C-terminal interlinker region (green). A specialized asparaginyl endopeptidease (AEP, butelase-1 (PDB code: 6DHI) effects head-to-tail cyclization of the Cter M domain of the precursor *in planta* and results in a mature CterM cyclotide (PDB code: 2LAM).

Pea-like albumin-1 genes are restricted to the tribe Faboideae, as evidenced by the lack of hits when albumin-1 prepropeptide sequences from *P. sativum* are used as queries in a tblastn search on all sequences exclusive of the Faboideae. The canonical albumin-1 gene structure in all taxa examined thus far consists of a signal peptide followed immediately by a b-chain peptide domain with a knottin fold, a short intervening sequence, and a ∼54 aa a-chain domain (Figure 6, Cter M precursor). Typical functions ascribed to the albumin-1 gene family include protein storage and defense through the potentially toxic b-chain. Their function as a toxin is exemplified in the *Pisum sativum* albumin-1 b-chain (Pa1b), a peptide that effectively kills weevils and select insects through inhibitory activity of insect vacuolar proton pumps (Jouvensal et al., 2003; Chouabe et al., 2011).

Interestingly, the loops between the cystine residues are similar in size and in some cases residue composition between *C. ternatea* cyclotides and other albumin-1 b-chains (Gilding et al., 2015). This observation implies that the development of cyclotide domains from progenitor albumin-1 b-chains would have involved adaptation of the b-chain into a cyclotide domain structure. A further necessary adaptation to facilitate cyclization is the acquisition of an Asp or Asn residue at the C-terminus of the cyclotide domain. These specific residues are required for cyclization by asparaginyl endopeptideases (AEPs) through a transpeptidation reaction between the C-terminal Asp/Asn residue and the N-terminal residue (Figure 6) (Nguyen et al., 2014; Harris et al., 2015).

In *C. ternatea*, all transcripts encoding albumin-1 a-chain domains contain a cyclotide domain in place of what would otherwise be the b-chain domain. The complete transition of this region in *C. ternatea* albumin-1 genes to cyclotide domains implies canonical b-chains were disfavoured in the evolutionary history of *C. ternatea* (Gilding et al., 2015). The albumin-1 gene family members of *C. ternatea* are ∼74 in number, whereas albumin-1 gene families from the other genome-sequenced Faboideae, *Glycine*, *Medicago*, and *Phaseolus*, are 3, 33, and 17 in number respectively (Goodstein et al., 2012; Gilding et al., 2015; Nguyen et al., 2016b). This observation on albumin-1 gene expansion in *C. ternatea* further supports the hypothesis that cyclotide domains exhibit qualities and functions that increase fitness.

Transcriptomic expression analysis of various *C. ternatea* organs illustrates the partitioning of cyclotide expression to certain organs for some precursors, while other precursors are expressed constitutively throughout the examined organs (Gilding et al., 2015). As a class of defense molecules, it is logical that some would be preferentially expressed to target specific threats that different organs may face. Other albumin-1 genes are expressed at a notable level throughout the whole plant. The precursor for Cter M is an example of a cyclotide that is expressed constitutively, so much so that transcripts encoding Cter M are upward of ninefold higher than the rubisco small subunit in shoots (Gilding et al., 2015). Clearly, the plant is investing large amounts of resources to produce these transcripts and the resulting peptides.

#### Peptide Characterization

*Clitoria ternatea* cyclotides generally have Gly residues at the proto- N-terminus and Asn residues at the proto- C-terminus of the cyclotide domain within the precursor proteins, similar to the case from other plant families. By contrast with the conserved terminal residues, the intervening backbone loops between the conserved Cys residues tend to be variable in size and sequence. Some of the biophysical properties of *C. ternatea* cyclotides deviate notably from peptides of other cyclotide-producing plant families. For example, Cter 13 contains eight Arg residues that confer a predicted charge of +7 and pI of 10, well above that predicted for MCoTI-I, which contains four Arg residues, from the Cucurbit *Momordica cochinchinensis* (Felizmenio-Quimio et al., 2001; Mylne et al., 2012). The more highly charged and high-pI cyclotides of *C. ternatea* are preferentially expressed in organs that encounter challenges from the soil, namely the roots and seeds of the plant. Cyclotide extracts from roots, compared to leaves, exhibit increased toxicity against the juvenile L1 stage of the model nematode *C. elegans*, whereas adults and late stage juveniles were not affected (Gilding et al., 2015). The high charge of these potentially nematicidal peptides in on trend with other described nematicidal peptides (Liu et al., 2011). Further study is required to test for specific activity of organ-specific cyclotides against organisms.

Cyclotide sequences observed in aerial tissue typically have lower predicted charges and pI values than cyclotides in soil-contacting tissues. Cyclotide extracts of these aerial tissues exhibit a different MALDI-MS profile compared to other plant parts and greater propensity to bind to insect-mimetic plasma membranes. This implies that the aerially-expressed cyclotides are targeting insects (Gilding et al., 2015).

The cyclotide diversity of *C. ternatea* is further increased by post-translational modifications (PTM). Serra et al. (2016) described the first observations of hexosylation and methylation of cyclotides through enzymatic digests and MS techniques, with the estimated cyclotide diversity conferred by primary sequence and PTM diversity numbering in the hundreds. What the function of these post-translational modifications may be remains to be defined. Modifications of amino acid side chains reported in cyclotides include oxidation (Met and Trp), methylation, deamination (common at C-terminal Asn to Asp), hexosylation, dehydration, and hydroxylation (select Pro residues) (Plan et al., 2007; Serra et al., 2016).

#### Biosynthetic Auxiliary Enzymes

Cyclotide transcripts of *C. ternatea* encode for a signal peptide that immediately precedes the N-terminal residue of the cyclotide domain (Poth et al., 2011a; Gilding et al., 2015; Nguyen et al., 2016b). The current model for *C. ternatea* cyclotide biosynthesis mimics that of other cyclotide producing species and begins with the signal peptide inducing the docking of the ribosome-transcript complex with the rough endoplasmic reticulum (ER) (Conlan and Anderson, 2011; Göransson et al., 2015). Unique to *C. ternatea* cyclotide precursors is that the signal peptide cleavage alone releases the N-terminus of the cyclotide domain, thus no other N-terminal processing proteases are required. Following this, it is postulated that folding of the cyclotide domain begins, presumably aided by protein disulfide isomerases (PDIs), as the propeptide is held within the ER. From there the folded propeptide is transported via vesicles to the Golgi, and later to prevacuolar or vacuolar compartments. Somewhere during this transport pathway the propeptide encounters a specific type of AEP that catalyzes the simultaneous cyclization and cleavage of the cyclotide domain from the precursor (Göransson et al., 2015; Jackson et al., 2018). Post-translational modifications are possibly acquired along the maturation pathway but are poorly defined and thus need further investigation (Serra et al., 2016).

##### Protein disulfide isomerases

The disulfide knot of cyclotides must be formed from the oxidation of the six cysteine residues in a specific order. Incorrect connectivity may result in the precursor not being able to be cyclized and flagged as a faulty molecule needing destruction. In all cyclotide producing taxa, the specific *in vivo* physical of genetic interactions of PDI family members and cyclotide precursors is not known. *In vitro* evidence for PDI involvement is known from a PDI cloned in the Rubiaceae plant, *Oldenlandia affinis*, however, under the conditions tested the isolated PDI was not as efficient as using an isopropanol buffer to effect proper disulfide bond formation (Gruber et al., 2007). A systematic *in vivo* examination of *C. ternatea* PDIs discovered in the transcriptome is hindered by the lack of reverse genetic resources in *C. ternatea*.

##### Asparaginyl endopeptidases

Asparaginyl endopeptidases (AEPs), like most proteases, are known primarily for their function in peptide bond hydrolysis (Yamada et al., 2005), thus a proposed role for peptide bond creation for a selection of AEPs is particularly intriguing. The first direct evidence for this came about through work by the Tam group (Nguyen et al., 2014), who set out to identify the peptide ligase responsible for the maturation of cyclotides in *C. ternatea*. Through activity-guided protein-fractionation studies, the researchers identified a single *C. ternatea* AEP isoform (termed butelase-1) that was highly efficient in intermolecular peptide cyclization. Since the discovery of butelase-1 in 2014, several other AEP peptide ligases have been identified from cyclotide producing plant species, including OaAEP1_b_ from *O. affinis* (Harris et al., 2015), PxAEP3b (*Petunia* x *hybrida*) (Jackson et al., 2018), and HeAEP3 (*Hybanthus enneaspermus*) (Jackson et al., 2018). Through bioinformatic and functional testing the structural features that differentiate AEP ligases from proteases are beginning to emerge. Specifically, plant AEPs that function as transpeptidase-preferring enzymes *in vivo* have been shown to possess specific markers in their protein sequence, most notably one termed the Marker for Ligase Activity (MLA) (Jackson et al., 2018).

Subsequent work by Gilding et al. defined the expression levels of butelase-1 (referred to as CtAEP1) and the full length sequence of butelase-2/CtAEP2, CtAEP3, and CtAEP5 via RNA-seq (Gilding et al., 2015). In contrast, a total of six butelases were described by Nguyen et al. (2014), with assembled sequences for butelase-4 and -6 not showing any homology to any of the CtAEPs described by Gilding et al. (2015). It might be the case that there is natural AEP isoform variation amongst *C. ternatea* accessions, or that differences in data assembly conditions, or choice of tissue RNA sampled between the studies of Nguyen et al. (2014) and Gilding et al. (2015) are responsible for this apparent discrepancy. Importantly, of all six AEPs, only butelase-1 has been shown to prefer transpeptidation over proteolysis.

## Next Generation Applications

In this section we describe recent applications of *C. ternatea* components in biotechnological, agricultural and pharmaceutical industries.

### Butelase

Butelase-1 has proven to be a very versatile molecular tool for a range of *in vitro* peptide engineering applications (Nguyen et al., 2016a,c; Bi et al., 2017). When compared to other characterized AEP ligases, butelase-1 displays superior reaction kinetics. Despite this, one obvious limitation for end-user uptake is that a recombinant production system is yet to be established (Nguyen et al., 2014). In lieu of this, a detailed protocol for the purification of butelase-1 from *C. ternatea* seed pods is available (Nguyen et al., 2016c), but is restricted to those with access to the source material and protein purification expertise. It remains unclear if butelase-1 has evolved superior structural features over other AEP ligases or that its greater catalytic efficiency is a by-product of purifying source activated enzyme.

#### Butelase-1 Mediated Intramolecular Peptide/Protein Cyclization

Tools to enable backbone cyclization of peptides have garnered considerable interest from the pharmaceutical industry as a means to provide proteolytically stable peptide therapeutics (Craik et al., 2012). In this regard, butelase-1 has been demonstrated as a highly versatile enzyme, cyclizing a range of diverse peptides, including cysteine rich cyclotides, conotoxins (e.g., MrIA) and sunflower trypsin inhibitors (SFTI-1) (Nguyen et al., 2014). Additionally a wide range of non-cysteine containing peptides have been cyclized, including human apelin, galanin, neuromedin U and salusin (Nguyen et al., 2015). In all cases, the substrate requirements for cyclization include the introduction of, if not already present, an Asn residue at the peptide ligation point, which must be linked to the C-terminal tailing residues of His-Val. These tailing residues, which are subsequently cleaved off and are not incorporated into the final cyclized product have been shown to be essential for butelase-1 cyclization efficiency (Nguyen et al., 2014). At the substrates N-terminus, requirements are flexible at the P1’ position, with all residues accepted apart from Pro. However, at the P2’ position more stringent requirements exist, with Cys, Ile, Leu, and Val all preferred (Nguyen et al., 2014). Together these requirements mean that most peptides, require at least some modifications of the termini residues to allow butelase-1 mediated cyclization. When these substrate requirements are met, butelase-1 has remarkably catalytic efficiency, with substrate to enzyme ratios of 100 ∼ 1000:1 commonly used, with typical cyclization reactions completed within 5 ∼ 30 min (Nguyen et al., 2015).

The benefits of backbone cyclization are not limited to small peptides, with the thermal and proteolytic stability of a number of larger proteins also improved by backbone cyclization. Like smaller peptides, these proteins must first be engineered to include optimal flanking residues for butelase-1 activity, with specific consideration given to the proximity of N and C residues. Where termini are not held close enough together, considerations should be given to include appropriate sized linker sequences. Using butelase-1, three different recombinantly produced proteins have been successfully cyclized, including green fluorescent protein (GFP), interleukin-1 receptor antagonist (IL-1Ra) and human growth hormone (somatropin) (Nguyen et al., 2015). In all cases butelase-1 (0.1uM) and target protein (25 μM) were incubated together with cyclization essentially complete within 15 min. In the case of IL-1Ra, backbone cyclization was shown to increase the thermostability of the protein, without affecting biological activity (Nguyen et al., 2015).

#### Butelase-1 Mediated Intermolecular Peptide Bond Formation

Butelase-1 has additionally shown great potential for the selective labeling of proteins by intermolecular peptide bond formation. Here, butelase-1 recognizes the required NHV motif engineered into a protein of interest and initiates ligation of incoming intermolecular nucleophiles, provided that substrate requirements are met. In this way a protein of interest can be labeled with any number of functional cargoes. Site specific labeling of proteins has applications for elucidating cellular pathways, defining protein–protein interactions and for the development of innovative medical imaging approaches and therapeutics (Falck and Müller, 2018; Harmand et al., 2018). One additional promising application is the site specific labeling of surface proteins of live bacteria (Bi et al., 2017). To accomplish this, the authors engineered an NHV motif to the C-terminus of the anchoring protein OmpA of *Escherichia coli*. Upon incubation of live cells with butelase-1, a range of cargo molecules were able to be successfully linked to the engineered bacterial surface protein OmpA. These included a fluorescein probe, useful for cellular tracking of pathogen response, and a tumor associated monoglycosylated peptide, which provided a proof of concept for delivering post translationally modified antigens as live bacteria vaccines.

### Insecticidal Applications of *C. ternatea* Peptide Extracts

Conventional pesticides have for decades been of paramount importance in sustaining agricultural productivity under an ever-increasing population burden. However, many traditional pesticides are increasingly becoming disfavored due to off-target toxicities and human health concerns. These concerns, together with increasing incidences of insects developing resistance mechanisms necessitates the discovery or engineering of novel pesticides with new modes of action (Perry et al., 2011). Recently an organic ethanolic extract prepared from *C. ternatea* vegetative tissue has shown promising insecticidal activity against a wide range of crop pests^3^. The extract, termed Sero-X^®^ has thus far been registered in Australia for applications in cotton and macadamia, with further applications pending both in Australia and overseas. Although the exact mode of action of this ethanolic extract remains to be determined, it is likely in part to be due to the high concentrations of *C. ternatea* cyclotides present (Poth et al., 2011a,b; Gilding et al., 2015). The prototypic *C. ternatea* cyclotide Cter M is indeed enriched in the Sero-X^®^ extract and when tested in isolation, displays lethality against cotton budworm (*H. armigera*) (Poth et al., 2011a). Like other cyclotides, such as kalata B1 from *O. affinis*, the predicted mode of action is through insect cell membrane disruption (Poth et al., 2011a; Craik, 2012), but it remains unclear if other non-proteinaceous components present in the Sero-X^®^ extract play a synergistic role. Importantly, Sero-X^®^ displays no toxicity to tested rodents or bee pollinators, and is considered non-hazardous according to the Globally Harmonized System of Classification and labeling of Chemicals.

### Food Colorants/Consumer Products

Butterfly pea flowers can range from white to intense blue to shades in between. This coloring largely stems from the anthocyanin content and degree of aromatic acylation (Kazuma et al., 2003a). The deep blue pigment of *C. ternatea* has been particularly popular in Asia, where flower petals are used to color teas, deserts and clothes. More recently, *C. ternatea* flower extracts have been used to create vibrant blue alcoholic gins^4^, which change color depending on the pH, such as occurs on mixing with tonic water or lime. Specifically, the deep blue color of *C. ternatea* flowers is a particularly sought after alternative to synthetic blue food colorants which have become increasingly disfavored due to health concerns (Nigg et al., 2011). Studies reported that addition of *C. ternatea* extracts increased the polyphenolic and antioxidant contents of sponge cakes (Pasukamonset et al., 2018), enhanced the oxidative stability of cooked pork patties (Pasukamonset et al., 2017) and reduced the predicted glycemic index of flour (Chusak et al., 2018a). Microencapsulation using alginate prevented the degradation and enhanced the retainment of the antioxidant activities of *C. ternatea* polyphenolic extracts post gastrointestinal digestion (Pasukamonset et al., 2016). Currently there exists no commercial scale production of *C. ternatea* for anthocyanins, with harvesting of plant material at large-scale not likely to be economically feasible. However, recent advances in engineering plant cell suspension cultures with anthocyanin regulatory pathway genes offers an alternative approach (Appelhagen et al., 2018).

## Conclusion and Future Outlook

Here we have attempted to provide a comprehensive and multidisciplinary account of the diverse properties and applications of *C. ternatea* and its constituent molecules. The plant is readily grown in a range of habitats and there is wide opportunity for it to be used for rotational cropping to aid in soil nitrogen regeneration, as a fodder crop for cattle, or as source of novel phytochemicals. There are already a host of cosmetic and food colorants on the market and the first *C. ternatea* based insecticide (Sero-X^®^) is also approved and being used for insect control on cotton and macadamia nut crops. The butelase-1 enzyme derived from *C. ternatea* pods is also creating a lot of interest as a biotechnological tool for peptide ligation and cyclization.

We anticipate that the success of products (including enzymes, extracts, and purified phytochemicals) deriving from *C. ternatea* will encourage more research on this plant and stimulate further discoveries that might lead to second and third generation products. For example, so far only a small fraction of the more than 70 cyclotides in this plant have been tested for pesticidal activity and there may be components in this cocktail of cyclotides that are significantly more potent as pesticides than what is currently known. Further work is needed to understand the biotic and abiotic factors that modulate the production of individual cyclotides in this plant and to understand possible synergies between different cyclotides and between cyclotides and non-cyclotide components.

We also anticipate that there will be more studies in the future on pharmaceutical applications of *C. ternatea* components. The ability to harvest large amounts of plant material means that one of the limitations encountered in many natural product research and commercialization (i.e., lack of source material) is not a factor for *C. ternatea*. While the multitude of medicinal applications reported so far from various *C. ternatea* preparations are impressive, we caution that many of these are one-off studies that have yet to be independently validated by groups other than the original reporting group. It is to be expected that the claims for the various bioactivities of plant extracts need to be tested with rigorous controls to establish the efficacy of the plant components. Furthermore, very few of the cyclotides in *C. ternatea* have been screened for medicinal applications and we feel this would be a useful exercise for future studies. Likewise, none of the *C. ternatea* cyclotides have yet been used as molecular grafting frameworks to introduce new desired pharmaceutical activities as has been done for cyclotides from other plants such as kalata B1 or MCoTI-II. With these suggestions for future work on this fascinating plant we feel that many more exciting discoveries are on the horizon.

## Author Contributions

DC and GO conceived and planned the framework for this article. All authors contributed to the writing and editing.

## Conflict of Interest Statement

The authors declare that the research was conducted in the absence of any commercial or financial relationships that could be construed as a potential conflict of interest.

## Footnotes

1 https://innovate-ag.com.au/

2 https://avh.chah.org.au/

3 https://innovate-ag.com.au/

4 https://www.inkgin.com/
